# Effect of Chitosan Properties and Dissolution State on Solution Rheology and Film Performance in Triboelectric Nanogenerators

**DOI:** 10.3390/gels11070523

**Published:** 2025-07-05

**Authors:** Francisca Araújo, Solange Magalhães, Bruno Medronho, Alireza Eivazi, Christina Dahlström, Magnus Norgren, Luís Alves

**Affiliations:** 1 University of Coimbra, CERES, Department of Chemical Engineering, Pólo II—R. Silvio Lima, 3030-790 Coimbra, Portugal; franciscaraujo1611@gmail.com (F.A.); solange.magalhaes@miun.se (S.M.); 2 Surface and Colloid Engineering, FSCN Research Centre, Mid Sweden University, SE-851 70 Sundsvall, Sweden; bfmedronho@ualg.pt (B.M.); alireza.eivazi@miun.se (A.E.); christina.dahlstrom@miun.se (C.D.); magnus.norgren@miun.se (M.N.); 3 MED—Mediterranean Institute for Agriculture, Environment and Development, CHANGE—Global Change and Sustainability Institute, Faculdade de Ciências e Tecnologia, Universidade do Algarve, Campus de Gambelas, Ed. 8, 8005-139 Faro, Portugal

**Keywords:** chitosan, acetic acid, solution viscosity, film formation, triboelectric nanogenerators

## Abstract

Chitosan films with potential application in triboelectric nanogenerators (TENGs) represent a promising approach to replace non-biobased materials in these innovative devices. In the present work, chitosan with varying molecular weights (MW) and degrees of deacetylation was dissolved in aqueous acetic acid (AA) at different acid concentrations. It was observed that the MW had a greater influence on the viscosity of the solution compared to either the acid concentration or deacetylation degree. Gel formation occurred in high-MW chitosan solutions prepared with low AA concentration. Films prepared from chitosan solutions, through solvent-casting, were used to prepare TENGs. The power output of the TENGs increased with higher concentrations of AA used in the chitosan dissolution process. Similarly, the residual AA content in the dried films also increased with higher initial AA concentrations. Additionally, hot-pressing of the films significantly improves the TENG power output due to the decrease in morphological defects of the films. It was demonstrated that a good selection of the acid concentration not only facilitates the dissolution of chitosan but also plays a key role in defining the properties of the resulting solutions and films, thereby directly impacting the performance of the TENGs.

## 1. Introduction

Triboelectric nanogenerators (TENGs) have gained significant attention in recent years due to their promising application in areas such as sensors, wearable electronics and biomedical devices [1]. For successful integration into these technologies, TENGs need to present suitable electrical performance, particularly high current density. Typically, these properties have been achieved through the use of composite materials or surface treatments [1]. However, most conventional TENGs rely on non-renewable polymers, like fluoropolymers and polydimethylsiloxane (PDMS), which implies challenges regarding sustainability and biodegradability.

To tackle these limitations, the development of bio-based TENGs is strongly advancing, since bio-based polymers offer distinct properties such as biodegradability, biocompatibility and natural abundance [2]. Notable progress has been made with cellulose-based TENGs, representing a great step toward greener alternatives [3]. Among other bio-based candidates, chitosan is a particularly promising polymer due to its electron-donating ability, which is highly advantageous for triboelectric performance [4].

Chitosan (poly β-(1→4)*N*-acetyl-d-glucosamine) is the deacetylated form of chitin [5], a major structural component in crustacean shells; it has long been treated as an industrial waste byproduct. In recent decades, chitosan has attracted considerable interest due to its favourable biological properties, such as its antimicrobial, antifungal, antitumour and hypocholesterolemic activities [6,7]. Moreover, the absence of toxicity and its excellent biodegradability and biocompatibility have expanded its use in biomedical and food packaging applications [8,9].

One of chitosan’s most valuable features is its ability to form high-quality films, which has led to its application in edible coatings and active packaging [8,10,11]. Chitosan is not soluble in pure water or in common organic solvents [12]. However, it can be solubilised in mildly acidic aqueous environments (typically below pH 6.0), where its amino groups (pKa ca. 6.5) are protonated, turning it from a nonionic polymer into a polyelectrolyte [13]. This transition in the protonation state enhances solubility through entropy-driven counterion gain.

Several organic and inorganic acids have been effectively used to dissolve chitosan, including acetic, formic, L-glutamic, lactic, hydrochloric and malic acid [14]. Organic acids are natural substances found in different fruits and fermented products, being usually preferred for chitosan dissolution, mainly targeting food applications [15]. Additionally, organic acids are preferred due to their lower corrosivity and suitability for biological applications [16].

To enhance the performance of bio-based polymers for applications in energy and materials science, chemical modifications are often employed [17,18]. For example, chemical pre-treatments have been performed in cellulose nanofibers (CNFs), where TEMPO-mediated oxidation and cationization can significantly improve nanofibrillation and dispersion, rheological behaviour and application-specific functionalities. For instance, TEMPO oxidation generates crystalline nanofibers with increased aspect ratios, producing tougher hydrogels at relatively low concentrations due to effective physical entanglements [19]. Cationization, particularly by introducing quaternary ammonium groups, imparts positive charges that increase antimicrobial activity and expand the potential applications of CNFs to areas such as water treatment and environmental remediation [20]. These strategies also enable fine-tuning of rheological behaviour [21].

While such chemical modifications have been extensively studied for nanocellulose, there remains a gap in understanding how the intrinsic molecular properties and dissolution behaviour of chitosan affect its rheology, film formation and performance in TENGs applications. Although several studies demonstrate that chitosan-based films can be functional in TENGs, their performance is often enhanced by incorporating functional fillers, such as BaTiO_3_, carbon nanotubes, or clay [22,23,24]. Yet, as stressed above, the fundamental relationship between chitosan’s molecular characteristics, solution rheology and triboelectric efficiency remains rather underexplored.

This study aims to address this knowledge gap by systematically evaluating how chitosan’s molecular weight, degree of deacetylation and dissolution pH influence its solution properties, film quality and triboelectric performance. By establishing clear links between the rheological profile of chitosan solutions and the performance of the resulting films in TENG applications, this work seeks to provide valuable insights for the development of optimised biopolymer-based TENGs, promoting sustainable and efficient energy-harvesting technologies for a wide range of applications.

## 2. Results and Discussion

### 2.1. Effect of Chitosan Molecular Weight

**Figure 1:**
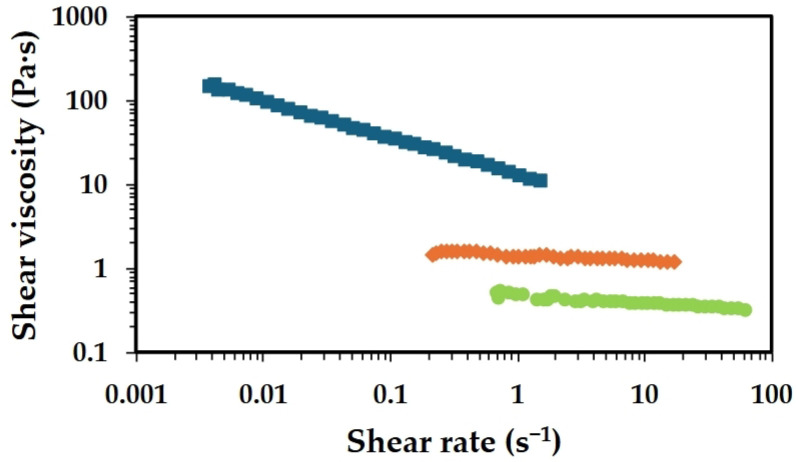
Flow curves of solutions containing 2.5 wt.% chitosan with different MWs in aqueous acidic media (2.0 wt.% AA), at 25 °C. Low MW (light green colour), medium MW (orange colour) and high MW (blue colour).

Figure 1 shows the flow curves of chitosan solutions of different molecular weights (MW), each dissolved in aqueous acidic media.

The high-MW chitosan solution consistently exhibits the highest viscosity across all shear rates, as expected. Additionally, a clear change in the rheological behaviour can be observed as the MW of chitosan increases from low to high. While the low- and medium-Mw chitosan solutions display a behaviour close to that of Newtonian fluids, a clear shear-thinning behaviour is observed for the high-MW chitosan sample. In shear-thinning systems, the polymer chains, initially entangled in the solution, align with the flow direction under increasing shear rates, reducing resistance and enhancing fluidity. This behaviour is commonly observed in polymer solutions and indicates that the fluid becomes more flowable as shear forces increase.

Polymers with higher molecular weight have longer chains, leading to more entanglements and stronger intermolecular interactions. These interactions create more structured networks, enhancing the resistance to flow and thus increasing the solution’s viscosity. Therefore, the high-MW solution behaves more like a highly viscous fluid, which is advantageous for applications where thicker fluids are required and typically results in more robust films. In contrast, the medium and low-MW chitosan solutions exhibit significantly lower viscosities. The medium-MW solution has a slightly higher viscosity than the low-MW one, though both remain well below the viscosity of the high-MW solution. This trend highlights the strong correlation between molecular weight, viscosity and overall rheological behaviour. It is important to highlight that the lower viscosities of the medium and low-MW solutions could be beneficial in certain processing scenarios, where lower flow resistance is preferred. Besides the MW range, polydispersity also plays a role in the rheological behaviour of chitosan solutions. Chitosan with low polydispersity results in solutions with higher zero-shear viscosity, while polydisperse samples result in a more gradual and broader relaxation of polymer chains [25]. The polydispersity index (PDI) reported for the commercial chitosan used in the present work ranges from 1.3 to 1.8, revealing chitosan’s relatively low polydisperse, which resulted in samples with high zero-shear viscosity, even at a moderate concentration.

The data obtained in the rotational tests can be fitted using rheological models such as the Power law or Ostwald–de Waele model (Equation (1)), within the measured shear rate range:

(1) $$\sigma =K\times {\dot{\gamma }}^{n},$$

where *K* is the consistency coefficient and *n* is the flow behaviour index. A value of *n* equal to 1 indicates a Newtonian behaviour; *n* < 1 corresponds to shear-thinning behaviour (pseudoplastic fluid); and *n* > 1 indicates a dilatant behaviour (shear-thickening fluid). For the low-MW chitosan, the values obtained for *K* and *n* were 0.48 Pa s^n^ and 0.90 (r = 0.986), respectively, whereas for medium-MW chitosan, *K* = 1.47 Pa s^n^ and *n* = 0.93 (r = 0.987). In contrast, the high-MW chitosan exhibited *K* = 15.19 Pa s^n^ and *n* = 0.58 (r = 0.997). The flow behaviour indices for the low- and medium-MW samples are close to 1, indicating behaviour near that of Newtonian fluids. Conversely, the high-MW chitosan solution clearly exhibits a shear-thinning behaviour, as indicated by an *n* value significantly below 1. The consistency coefficient increases markedly as the MW of chitosan increases, which is consistent with the presence of longer molecular chains and more entangled networks for high-MW systems.

### 2.2. Effect of Chitosan Concentration

**Figure 2:**
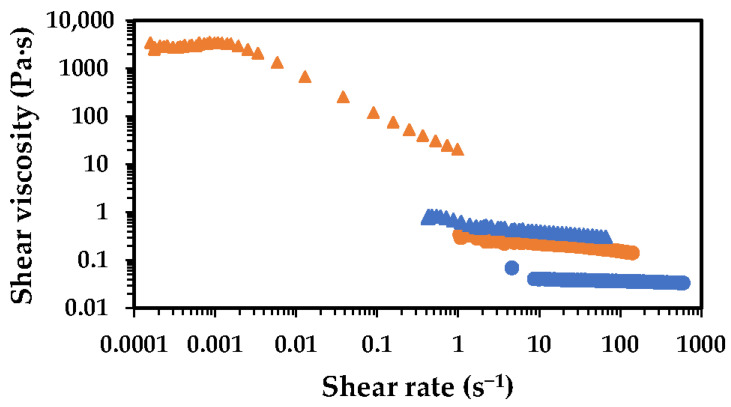
Flow curves showing the effect of chitosan concentration (in 5% aqueous acetic acid) on the shear viscosity of the solutions at 25 °C. Low-MW chitosan is represented by blue symbols, while the high-MW chitosan is represented by the orange symbols. Solutions containing 1.0 wt.% chitosan are shown as circles, while those with 2.5 wt.% are shown as triangles.

The increment of chitosan concentration, from 1.0 to 2.5 wt.%, resulted in higher viscosity values, which were much more pronounced for the high-MW chitosan than the low-MW one (Figure 2). These changes in viscosity were accompanied by noticeable changes in the rheological behaviour, particularly for the high-MW chitosan. At 1.0 wt.%, the high-MW chitosan solution behaves closely to a Newtonian fluid, whereas at 2.5 wt.% it exhibits clear pseudo-plastic behaviour (shear-thinning). These differences in viscosity and flow behaviour of chitosan solutions are deeply related to the MW of the polymer, with the high MW being more prone to form entanglements with the neighbouring molecules, compared to the low MW at a certain concentration. On the other hand, high-MW chitosan is expected to reach the overlap concentration (C*) at lower concentrations compared to low-MW chitosan. Data from the literature indicate that chitosan with a molecular weight of ca. 130 kDa has a C* of about 0.07 wt.%, while high-MW chitosan (ca. 650 kDa) exhibits a C* around 0.038 wt.%. In the present study, the concentrations used, even the lowest one (i.e., 1.0 wt.%) exceed the C* for both chitosan types (low and high MW) [26]. This molecular weight-dependent transition is also expected to influence the mechanical properties of the resulting films, as longer chains in high-MW chitosan enable the formation of more robust and entangled networks.

### 2.3. Effect of Acetic Acid Concentration

**Figure 3:**
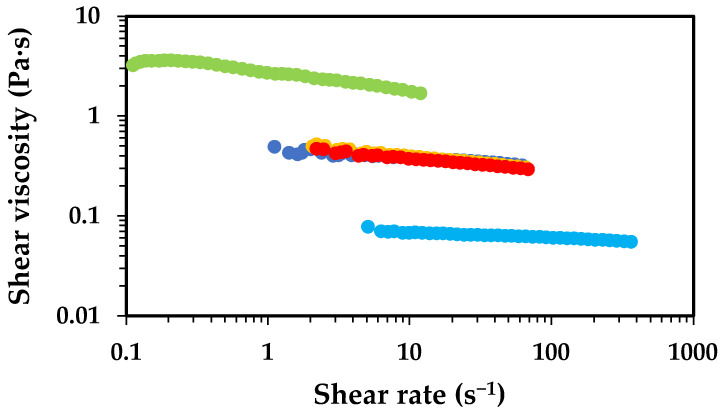
Effect of acetic acid concentration on the shear viscosity of solutions of low-MW chitosan (2.5 wt.%), at 25 °C. Solutions containing 0.5 wt.% AA (light green colour), 1.0 wt.% AA (light blue colour), 2.0 wt.% (blue colour), 5.0 wt.% (yellow colour) and 10.0 wt.% (red colour).

**Figure 4:**
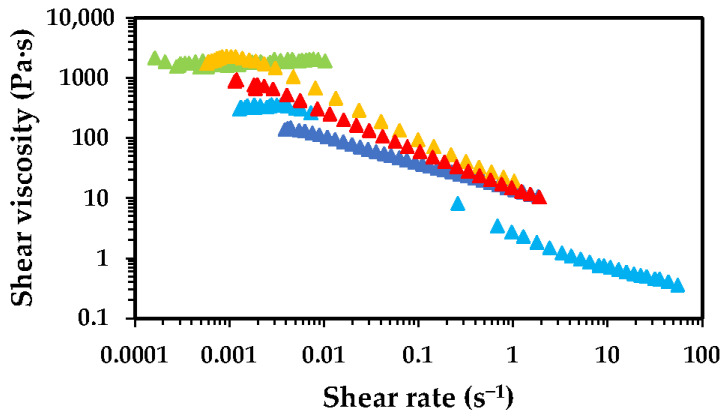
Flow curves showing the effect of acetic acid concentration on the shear viscosity of high-MW chitosan solutions (2.5 wt.%), at 25 °C. Solutions were prepared with 0.5 wt.% AA (light green symbols), 1.0 wt.% AA (light blue symbols), 2.0 wt.% (dark blue symbols), 5.0 wt.% (yellow symbols) and 10.0 wt.% (red symbols).

As mentioned above, the dissolution of chitosan in aqueous media occurs via protonation of its amine groups in the presence of acids, which transforms the nonionic polymer into a cationic polyelectrolyte. This protonation significantly enhances aqueous solubility due to the entropy gain from the release of counterions that neutralise the polymer charges [27]. The most common acids used for this purpose are acetic acid, lactic acid and hydrochloric acid [28]. It is anticipated that both the type and concentration of acid used have impact on the solubility and rheological properties of its solutions. To elucidate the effects of the concentration of AA and chitosan MW on the dissolution behaviour and rheological properties, Figure 3 and Figure 4 present the flow curves obtained for low- and high-MW chitosan for aqueous solutions containing different concentrations of AA.

The shear viscosity of the solutions changes as the AA concentration varies, with a similar trend observed for low- and high-MW chitosan solutions. The solutions containing 0.5 wt.% AA exhibit the highest viscosity, followed by a significant decrease in viscosity for the solutions containing 1.0 wt.% AA. Further increases in AA concentration led to a gradual rise in viscosity. The magnitude of the viscosity for low- and high-MW chitosan dissolved in 0.5 wt.% AA presents significant differences, with the high-MW sample reaching values of ca. 2000 Pa·s, approximately three orders of magnitude higher than the low-MW counterpart under the same conditions. This difference in viscosity is attributed to the longer polymer chains, which at lower AA concentrations may be only partially dissolved, due to incomplete protonation of amine groups. These partially dissolved chains tend to form aggregates capable of reinforcing the polymer network, resulting in solutions of higher viscosity. The pKa of chitosan has been reported to be dependent on MW and degree of deacetylation, typically ranging between 6.17 and 6.51; experimental data showed that decreasing the MW or increasing the deacetylation degree lowers the pKa [29]. The pKa of chitosan was shown to slightly decrease from 6.51 to 6.39 as the molecular weight decreased from 1370 to 60 kDa. In contrast, the degree of deacetylation had a more pronounced effect on pKa values, which increased from 6.17 to 6.51 as the degree of deacetylation decreased from 94.6% to 73.3%. It has been hypothesised that the degree of deacetylation influences the balance between hydrophobic interactions and hydrogen bonding on chitosan, thereby exerting a greater impact on pKa than molecular weight [29]. Therefore, under identical dissolution conditions (e.g., 0.5 wt.% AA), the high-MW chitosan is expected to have a lower number of protonated amines than low-MW chitosan, which aligns with the observed viscosity trends. The results obtained in the rotational tests were fitted using the Ostwald–de Waele rheological model (Table 1).

The data fitting shows different trends for low- and high-MW chitosan. In the case of low-MW chitosan, a slight increase in the consistency coefficient (*K*) was observed as the concentration of AA increased. Conversely, for high-MW samples, the K parameter generally decreased as the AA concentration increased, except for the solution containing 5.0 wt.% AA. Regarding the flow behaviour index (*n*), solutions prepared with low-MW chitosan tended to behave more like Newtonian fluids. In contrast, high-MW chitosan solutions exhibited pseudo-plastic (shear-thinning) behaviour, except for the sample prepared with 0.5 wt.% AA, which behaved as a Newtonian fluid within the studied shear stress range. For all other high-MW chitosan samples, pseudo-plasticity increased with rising AA concentration. These results are in line with those reported in the literature [30]. These differences in behaviour are intimately connected with the longer polymer chains in high-MW chitosan, able to form a 3D network which is disrupted by the increase in the shear stress applied (typical behaviour of a polymeric solution). In contrast, low-MW chitosan does not form a strong network, thus behaving closer to an ideal Newtonian fluid.

**Figure 5:**
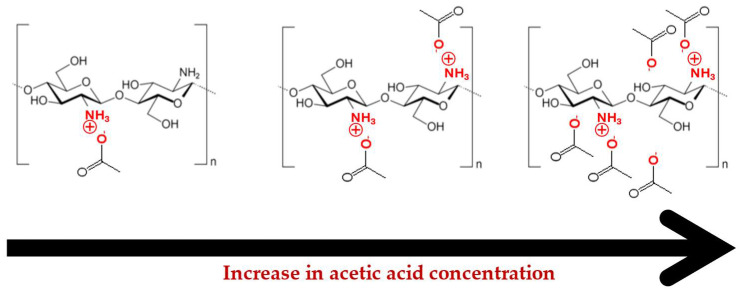
Schematic illustration of the effect of acetic acid concentration on the ionisation of NH_2_ groups and counterion interaction.

The formation of a strong 3D network and gel-like structure in high-MW chitosan dissolved in 0.5 wt.% AA is likely related to the partial ionisation of the polymer. As the acid concentration is raised, complete ionisation is obtained. The decrease in viscosity as the acid concentration goes from 0.5 wt.% to 1.0 wt.% can be attributed to enhanced ionisation of NH_2_ groups, improving solubility and reducing aggregation (especially noticeable at 1.0 wt.% AA). This is supported by zeta potential data which demonstrates that the positive charge of commercial chitosan (high MW) is near by 0 mV at pH 6.0, rises to ca. +30–40 mV at pH 5 (ca. 1.0 wt.% acetic acid) and reaches +60.0 mV at pH 4 (>2.0 wt.% acetic acid) [10,31]. However, at higher AA concentrations (5.0 and 10.0 wt.%), viscosity rises again. This is likely due to charge neutralisation effects caused by excess acetate ions (see Figure 5). Once full ionisation is achieved (apparently at around 1.0 wt.% AA, chitosan has above 92% NH_3_^+^ [31]), the number of counterions exceeds the number of positive charges on the polymer. This excess of negative ions may slightly reduce polymer solubility through a salting-out effect. Lower additions of AA promote protonation of the amine groups, turning chitosan into a polyelectrolyte and counterion dissociation (forming acetate ions). This process enhances solubility mainly due to an increase in entropy (and a lower but still synergistic decrease in enthalpy due to the increase in chitosan-water interactions). However, when the AA concentration exceeds approximately 0.3 M (~2.0 wt.% AA), the entropic gain is lost. The electrostatic repulsion among chains decreases, leading to their self-association into larger transient structures, resulting in an increase in solution viscosity. When added to aqueous solution, AA dissociates according to its acid dissociation constant (Ka = 1.8 × 10^−5^). Increasing the concentration of AA leads to the formation of more acetate and H^+^ ions (for simplicity, we use the Arrhenius definition of acids). This occurs up to the point where chitosan is fully protonated (most of H^+^ are used to protonate the amine groups) while acetate ions act as counterions. Beyond this point (ca. 0.3 M of AA), excess ions are present in solution, as not all are required for chitosan protonation. It is well known that different ions present different affinities to polymers or proteins, as described by the Hofmeister series. H^+^ ions combine with water to form H_3_O^+^, reducing the pH, whereas acetate ions, being less hydrophilic, can adsorb/interact with the surface of the polymer (chitosan). This reduces electrostatic repulsion among the polymer chains and promotes polymer aggregation. This phenomenon was clearly observed and rationalised by, for instance, Salaün et al., who reported that the screening of amino-charged chitosan chains with anions such as CH_3_COO^−^ and Cl^−^ increases the tendency of flocculation or precipitation of chitosan. Therefore, the increase in ionic strength (>0.46 M) enhances the aggregation by chitosan-chitosan attraction over the chitosan-solvent interaction and influences chitosan solubility. Chitosan in acidic medium shows an expanded conformation structure since the amino groups exert a repulsive force on each other, but the addition of salt or an increase in ionic strength shrinks the structure by increasing chain flexibility [32]. As a result, the occupied volume of chitosan chains in solution is reduced by increasing the ionic strength and a decrease in the intrinsic viscosity of chitosan solution is observed [33]. In addition, hydrophobic interactions of methyl groups of chitosan also contribute to self-association of polymer chains, which are more relevant when the electrostatic repulsion is screened by the excess of acetate ions [34,35].

### 2.4. Deacetylation Effect

**Figure 6:**
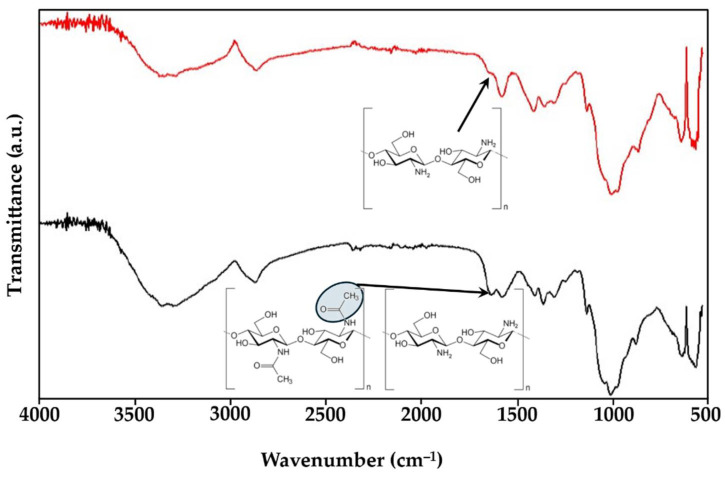
FTIR of chitosan (medium MW) with low deacetylation degree (black line) and high deacetylation degree (red line).

**Figure 7:**
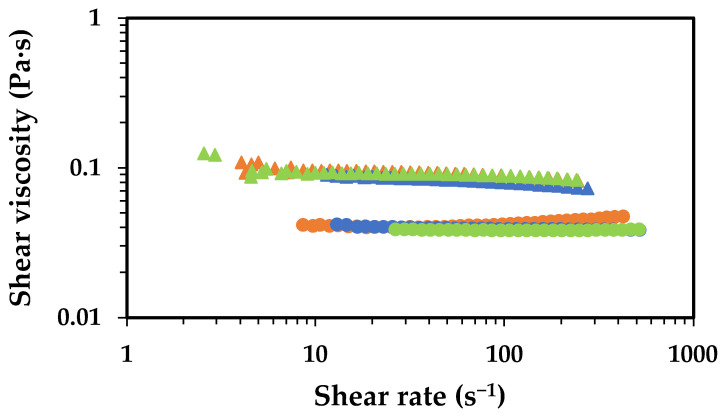
Flow curves showing the effect of chitosan deacetylation on the shear viscosity at 25 °C. Data regarding solutions containing 1.0 wt.% high deacetylated degree chitosan (circles) and 1.0 wt.% commercial chitosan (triangles), prepared with 2.0 wt.% AA (orange symbols), 5.0 wt.% AA (blue symbols) and 10.0 wt.% (light green symbols) are displayed.

The commercial chitosan used in the present work has a deacetylation degree of ca. 74%, which is in good agreement with the specification provided by the supplier (≥75%). After an additional deacetylation step, the chitosan achieved a very high content of free NH_2_ groups (98%). The success of the deacetylation was confirmed by FTIR (Figure 6), which shows a clear decrease in the band at ca. 1650 cm^−1^, attributed to the C=O bond stretching vibration in the acetyl groups of commercial chitosan [36]. After the additional deacetylation, this band is no longer present, confirming the success of the deacetylation process. It is expected that the number of free NH_2_ groups will have an impact on the solubility of chitosan and, consequently, on the rheological properties of its solutions. The flow curves of commercial and highly deacetylated chitosan are presented in Figure 7.

The increase in the number of free NH_2_ resulted in solutions of lower viscosity for all the AA concentrations tested. This effect can be attributed to the higher solubility of chitosan with a higher number of free NH_2_ groups, which, at the same pH, will have more protonated amines (NH_3_^+^). This higher cationic charge density renders the polymer into a more positive polyelectrolyte, thereby enhancing its solubility. The higher charge density induces higher electrostatic repulsion between the polymer chains, which results in reduced aggregation and, consequently, lower viscosity. Another possible explanation lies in a slight decrease in MW of chitosan caused by the harsh conditions of the deacetylation process (50.0 wt.% NaOH and 70 °C for 2 h). Based on the rheological data, the latter hypothesis seems more reasonable because the solutions’ viscosity remains basically unchanged regardless of the AA concentration used. If the primary factor were the increased charge density, one would expect a decrease in viscosity with increasing AA concentration. Rasweefali et al. (2021) reported the decrease in molecular weight of chitosan with the increase in deacetylation time [37]. Another support for the latter hypothesis comes from the work presented by Rinaudo et al. (1993) [38] in which demonstrated that the chain expansion of chitosan is relatively independent of the degree of deacetylation. This, an increase in the deacetylation degree, is not expected to produce significant changes in viscosity, but the possible slight decrease in the MW of chitosan occurred during the deacetylation process [38].

### 2.5. Oven Drying Time Effect

**Figure 8:**
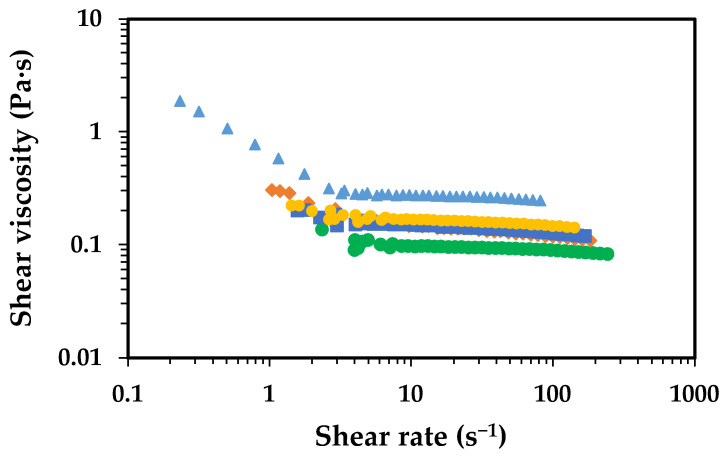
Flow curves showing the shear viscosity of medium-MW chitosan (1.0 wt.%) in acidic aqueous solution (2.0wt.% acetic acid) during film drying in the oven at 50 °C: 0 h (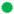), 1 h (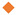), 2 h (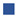), 4 h (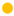) and 8 h (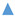).

During the film-forming process, the rheology of the solutions is expected to change significantly due to the polymer concentration (by solvent evaporation). In the case of chitosan solutions with organic acids, the concentration of the acids varies during the drying process. Figure 8 shows the results of the shear viscosity of medium-MW chitosan (1.0 wt.%) dissolved in aqueous solutions containing 2.0 wt.% AA.

The viscosity of the chitosan solutions increased with drying time during the first 8 h of oven drying. A similar behaviour and trend were observed for medium-MW chitosan solutions prepared in 10.0 wt.% AA. The decrease in the AA concentration is more pronounced for the sample initially containing 10.0 wt.% AA than for the sample with 2.0 wt.% during the earlier stages (1 h and 2 h). However, after 8 h, both acid concentrations were reduced to ca. 50% of the initial concentration (i.e., ca. 1.0 wt.% AA for the 2.0 wt.% sample and 5.0 wt.% for the sample containing 10.0 wt.% AA in the beginning). Despite this, the increase in viscosity was higher for the samples containing 2.0 wt.% AA than for the 10.0 wt.% samples. These results suggest that the rise in viscosity during drying is more closely related to the reduction in acetic acid content through evaporation than to an increase in polymer concentration alone. The lower acid concentration likely decreases chitosan solubility due to reduced protonation, promoting polymer aggregation and consequently increasing viscosity. This effect, combined with a slight increase in polymer concentration from solvent evaporation, led to a more pronounced viscosity increase in the sample with lower initial AA concentration.

### 2.6. Characterisation of Chitosan Films

**Figure 9:**
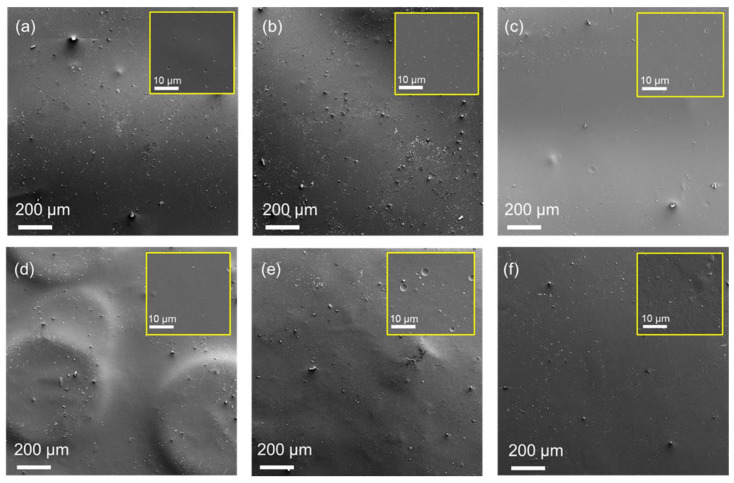
SEM surface images of (**a**) low MW, 2%, (**b**) low MW, 5%, (**c**) low MW, 10%, (**d**) high MW, 2%, (**e**) high MW, 5%, and (**f**) high MW, 10%, at 100× magnification. The inserted images with yellow frames correspond to each sample at 2 k× magnification.

The SEM images in Figure 9 reveal that the samples’ surfaces have non-uniformities, appearing as particles and air bubbles that formed craters of different sizes depending on the AA concentration.

**Figure 10:**
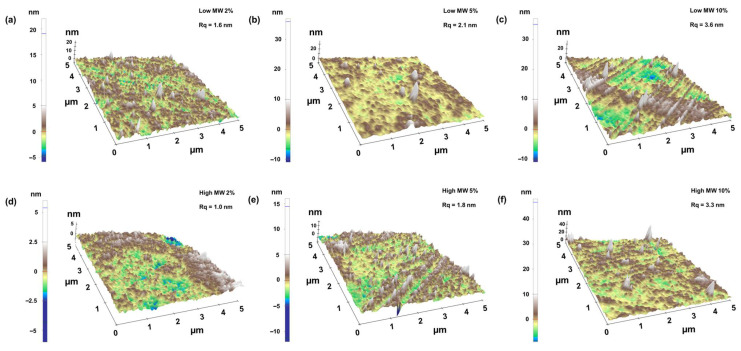
AFM 3D height images of chitosan films prepared with low- and high-MW chitosan dissolved in 2, 5 and 10% acetic acid solutions. (**a**) low MW, 2%, (**b**) low MW, 5%, (**c**) low MW, 10%, (**d**) high MW, 2%, (**e**) high MW, 5% and (**f**) high MW.

To further investigate the surface properties, AFM was employed to investigate the effect of chitosan MW and solvent acid concentration on the surface structure of the chitosan films (Figure 10). The AFM analyses and the calculated roughness (Rq) reveal that all the samples are relatively smooth (i.e., Rq ranging from 1.0 to 3.6 nm). However, films prepared with higher MW chitosan at varying acid concentrations were slightly smoother than those made from low-MW chitosan under comparable conditions. A closer inspection of AFM data shows that enhancing the acid concentration increases the surface roughness of the films made from both low- and high-MW chitosan.

**Figure 11:**
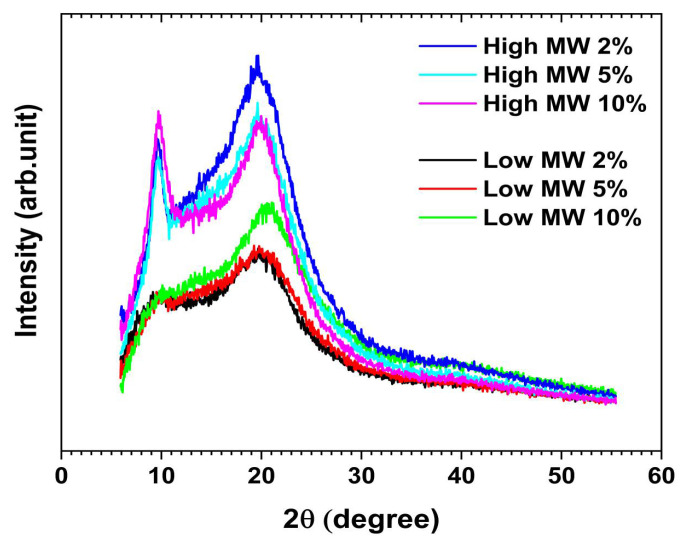
X-ray diffraction (XRD) patterns of the chitosan films fabricated from low and high-MW chitosan dissolved in 2, 5 and 10 wt.% acetic acid solutions. The intensity values were only subtracted from the blank run intensity of the sample holder and presented without applying the smoothing algorithm.

Chitosan is a semicrystalline polymer with various allomorphs that differ in the arrangement of the chains within their crystalline regions [39]. It has been observed that the structure of chitosan films can be influenced by the type of acid used for dissolution [40]. However, to the best of our knowledge, there are no systematic investigations regarding the combined effect of chitosan MW and solvent acid concentration on the structural features of chitosan films. In this regard, the X-Ray diffraction (XRD) analysis was performed on the chitosan films formed with low- and high-MW chitosan dissolved in 2, 5 and 10 wt.% AA solutions (Figure 11). The diffractogram peaks are typical of chitosan, with notable reflections at diffraction angles (2θ) around 10°, 14° and 20°, corresponding to the (010), (100) and (020) crystallographic planes, respectively [41].

Figure 11 clearly shows that the intensity of the (010) diffraction peak increases in films prepared with high-MW chitosan under similar conditions, indicating a higher degree of crystallinity. It is worth noting that increasing the film thickness in high-MW chitosan samples (i.e., 46–61 µm) enhances the intensity of the peaks compared to the thinner films prepared from low-MW chitosan (i.e., 36–46 µm).

The effect of AA concentration was also evaluated. The XRD patterns of films prepared from low-MW chitosan in 2 and 5 wt.% AA were nearly identical. However, a considerable increase in the intensity of the (020) diffraction peak (2θ ~20°) was observed in films prepared from 10 wt.% AA solutions. In contrast, films fabricated with high-MW chitosan dissolved in 10 wt.% AA, exhibit an increase in the (010) reflection peak and a decrease in the (020) peak relative to those prepared in 2 wt.% AA. For high-MW chitosan, film samples prepared from 5 wt.% AA solutions showed diffraction peak intensities for (010) and (020) that were similar to those prepared with 2 and 10 wt.% AA, respectively.

### 2.7. TENG Performance of the Chitosan Films

**Figure 12:**
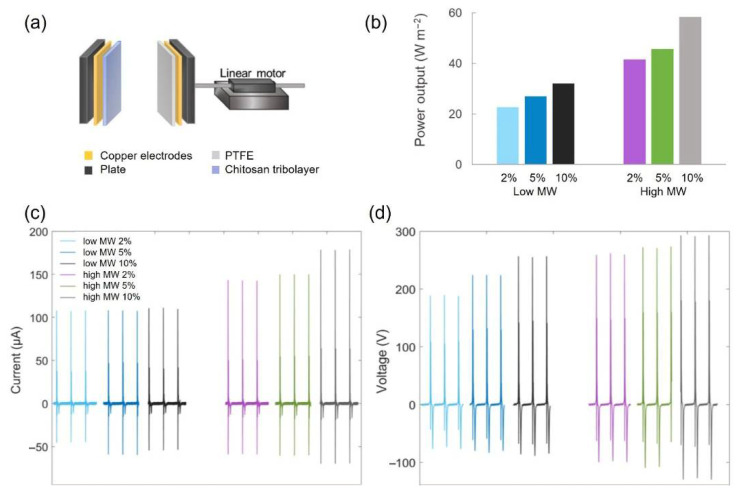
Triboelectric assays: (**a**) Schematic setup of TENG. (**b**) Power output of chitosan samples with low- and high-MW, prepared with increasing AA concentrations: 2 wt.%, 5 wt.% and 10 wt.%. Corresponding current (**c**) and voltage (**d**) profiles for the same samples.

The triboelectric measurements were performed using the contact-separation mode, and Figure 12a shows the TENG setup. In contact-mode TENGs, several parameters can influence the output voltage and current, specifically when polymeric layers with different electron gain or loss abilities come into contact. The parameters can be assorted into material and structural properties as well as operational conditions. In this regard, several strategies have been suggested to improve the TENGs performance, including surface modification, compositing, optimisation of the synthesis and processing conditions as well as the incorporation of functional groups [42].

Figure 12b shows the peak power output of chitosan films with low- and high-MW. The samples made with high-MW chitosan had a significantly higher power output. This enhancement is particularly noteworthy given that the high-MW samples (thickness: 46–61 µm) were thicker than those from low-MW chitosan (thickness: 36–46 µm), as increased film thickness typically leads to lower power output. Figure 12c reveals that the current remains rather constant for the low-MW samples, whereas the high-MW samples exhibit an increasing trend with increasing AA concentration. Both the low-MW and the high-MW samples show increasing voltage with the AA concentration (Figure 12d); notably, the high-MW sample prepared with 10 wt.% AA showed a substantial increase in current and voltage, resulting in 82% increase in power output compared to the 2 wt.% AA condition. Table 2 shows the triboelectric data, including standard deviation from 10 peaks of each measurement.

In our previous work [3], we identified that surface structure, mechanical properties influencing effective contact area and crystallographic orientation significantly govern the triboelectric performance of semicrystalline regenerated cellulose. In the present work, considering the semicrystalline structure of the chitosan samples (Figure 11), the enhanced triboelectric effect observed in high-MW chitosan films can be attributed to the more pronounced (010) reflection peak. The effect of surface roughness on the triboelectric response is likely negligible, as the samples are very smooth and show similar roughness values (e.g., Rq = 1.0–3.6 nm), according to AFM analysis. Quantification of residual AA in our cast films revealed acid contents ranging from 7% to 15% (relative to the total weight of the dried film), with the highest residual levels observed in films prepared from 10.0 wt.% AA solutions. No significant difference in residual AA content was found for low- and high-MW chitosan films. These residual acid levels may help explain the differences in the triboelectric performance observed with varying AA concentrations during chitosan dissolution and casting: higher voltage and current values were associated with higher residual AA content. A recent study further supports this, showing that the presence of carboxyl functional groups in carboxymethyl and other carboxylated chitosan derivatives enhances triboelectric output compared to unmodified chitosan [43].

## 3. Conclusions

The present study explored the effect of chitosan MW and AA concentration on the rheological behaviour and TENGs performance of chitosan-based films. It was concluded that both the MW of chitosan and the AA concentration significantly impact the rheological properties of chitosan in aqueous solution. High-MW chitosan forms solutions with higher viscosity, for the same concentrations of chitosan and AA, and can form gels at lower AA concentrations. An initial increase in AA concentration (up to ~1.0 wt.%) led to a decrease in viscosity, attributed to enhanced chitosan solubility. However, at AA above 2.0 wt.%, the viscosity increased again, likely due to reduced electrostatic repulsion and entropy of the system, which promoted self-association of chitosan chains into larger transient structures, which consequently increased solution viscosity.

Chitosan MW and AA concentration also showed a notable impact on the triboelectric properties of the films. High-MW chitosan and higher AA concentrations led to TENGs with higher output. High-MW chitosan resulted in more crystalline films, particularly with an intensified (010) reflection peak, which is associated with improved mechanical properties and enhanced triboelectric performance. Additionally, higher AA concentrations during dissolution led to increased levels of residual AA in the dried films. This residual AA significantly affected triboelectric performance, likely due to the elevated surface potential conferred by its presence.

This study provides valuable insights into how the molecular weight of chitosan and the concentration of acetic acid during processing influence the rheological and triboelectric properties of chitosan-based films. These findings not only deepen the fundamental understanding of chitosan’s behaviour in solution and solid-state but also pave the way for its more effective application in energy harvesting devices such as TENGs. By tuning molecular and processing parameters, it is possible to optimise film performance for specific functional needs. Future work should focus on systematically exploring other biocompatible acids and crosslinkers, evaluating long-term stability and performance under mechanical stress, and integrating these films into flexible or wearable TENG systems. Furthermore, correlating film crystallinity and surface chemistry with real-time TENG output may yield predictive models for performance optimisation across different biopolymer systems.

## 4. Materials and Methods

### 4.1. Materials

Chitosan (deacetylation ≥ 75%) and MW of ca. 60–190 kDa (low MW), ca. 190–310 kDa (medium MW) and ca. 310–375 kDa (high MW) were purchased from Sigma-Aldrich (Merck), Algés, Portugal. The polydispersity index (PDI) reported for medium-MW chitosan from Sigma-Aldrich is 1.3 (MW is 217 kg/mol and MN is 165 kg/mol). The PDIs for chitosans of different MW ranges (i.e., low, medium and high) have been reported to vary between 1.3 and 1.8 [44]. Acetic acid (glacial, >99.7% purity) was acquired from Sigma-Aldrich (Merck), Algés, Portugal. Sodium hydroxide pellets (>97% purity) were obtained from VWR (Carnaxide, Portugal). Hydrochloric acid (0.1 M aqueous solution) was acquired from Sigma-Aldrich (Merck), Algés, Portugal. The chemicals were used without further purification. All the samples were prepared using deionised water.

### 4.2. Methods

#### 4.2.1. Preparation and Characterisation of Highly Deacetylated Chitosan

The commercial chitosan (>75% deacetylation) was further deacetylated to produce highly deacetylated chitosan (>90%). To do so, ca. 5 g of medium-MW chitosan was dispersed in a 50 wt.% aqueous solution of NaOH and heated for 2 h at 70 °C. After the reaction, chitosan granules were extensively washed with deionised water until neutral pH. The granules were dried in an oven at 50 °C and stored. The deacetylation degree was estimated by acid-base titration. Briefly, chitosan (ca. 0.3 g) was weighed and dissolved in 30 mL of 0.1 M HCl at room temperature. After complete dissolution, two drops of methyl orange indicator were added, and the titration was then performed using a 0.1 M NaOH solution (previously standardised) until the colour of the solution changed from pink to orange. The NH_2_% and free NH_2_% were calculated following the work of Yuan et al. using the following equations [45,46]:

(2) $${NH}_{2}\%=\frac{\left[\left(C1V1-C2V2\right)\times 0.016\right]}{G(100-W)}\times 100,$$

(3) $$Free\;{NH}_{2}=\frac{{NH}_{2}\%}{9.94\%}\times 100$$

where *C*1 is the concentration of HCl (in M), *C*2 is the concentration of NaOH (in M), *V*1 is the volume of HCl (in mL), *V*2 is the volume of NaOH used (in mL), *G* is the weight of chitosan used, *W* is the chitosan sample water content (determined at 105 °C for 12 h), 0.016 corresponds to the NH_2_ content in 1 mL of HCl 1.0 M, and 9.94% is the theoretical content of NH_2_ (calculated by the ratio of the equivalent weight of NH_2_ (16 g mol^−1^) divided by the molecular weight of the chitosan monomer (161 g mol^−1^).

The deacetylation of chitosan was further confirmed by FTIR-ATR spectroscopy. A small amount of dried highly deacetylated chitosan, as well as commercial chitosan, were placed over the ATR crystal and the IR spectrum was recorded in the range of 4000–500 cm^−1^, at a resolution of 4 cm^−1^ and 128 scans.

#### 4.2.2. Preparation of Chitosan Solutions and Films

Solutions of chitosan of different MWs, as well as highly deacetylated chitosan, were prepared by dissolving precise amounts of polymer in acetic acid aqueous solutions, at concentrations ranging from 0.5 wt.% to 10.0 wt.%. Initially, the chitosan was dispersed in distilled water; once the polymer was well dispersed, the appropriate amount of acetic acid was added. The mixtures were then stirred using a magnetic stirrer until a homogeneous solution was obtained. After complete dissolution, chitosan solutions were centrifuged at 2200× *g* for 10 min to remove any residual undissolved polymer and air bubbles. Approximately 20 g of each chitosan solution was poured into plastic Petri dishes (90 mm in diameter) and cast into films by drying in an oven at 50 °C overnight.

#### 4.2.3. Rheology of Chitosan Solutions

The rheological measurements were performed in a stress-controlled rheometer RheoStress 1 (Haake, Model RS1, Karlsruhe, Germany) at constant temperature (25 °C) using a cone-plate geometry (C60/1Ti) connected to a temperature control recirculation bath (Haake Phoenix II, Karlsruhe, Germany). Flow curves were obtained in controlled stress mode, applying shear stresses ranging from 0.2 Pa to 20.0 Pa. The power law parameters, fitted using the Ostwald–de Waele model, were obtained using the Haake RheoWin 4.20.005 software (Haake, Karlsruhe, Germany).

#### 4.2.4. Characterisation of Chitosan Films

##### High-Performance Liquid Chromatography for the Determination of Residual Acetic Acid in Films

To quantify the residual AA present in the films after the casting process, small pieces of chitosan films were cut (ca. 50 mg) and placed in miliQ water (30 mL) under stirring overnight to allow the release of residual AA from the films. The extracted AA was then quantified by HPLC (Knauer, Berlin, Germany) equipped with a refractive index (RI) detector. The operating conditions in all HPLC analyses were as follows: injection volume of 20 μL; Rezex ROA-Organic acid column maintained at 40 °C with the respective guard column at room temperature; mobile phase with a flow rate of 0.6 mL/min, and RI detection at room temperature. The mobile phase used consisted of a 0.0025 M H_2_SO_4_ solution previously filtered with a 0.2 μm nylon membrane filter (Fioroni, Forlì, Italy). Calibration curves were prepared using standard solutions of AA.

The same equipment and analytical conditions were used for the quantification of AA during the drying process of the films, at different drying times (1 h, 2 h, 4 h and 8 h).

##### Scanning Electron Microscopy (SEM)

Field emission scanning electron microscopy (FE-SEM) images were obtained of the chitosan films using a TESCAN MAIA3 electron microscope (TESCAN, Brno – Kohoutovice, Czech Republic in secondary electron (SE) mode using 3 kV accelerating voltage and ~6mm WD. The chitosan samples were coated with a 2 nm Iridium layer using a Quorum Q150T ES (Quorum, East Sussex, UK) before image acquisition.

##### X-Ray Diffraction Analysis

X-ray diffraction (XRD) was performed at room temperature using a Bruker D2 Phaser diffractometer (Bruker, Billerica, MA, USA) with Cu Kα radiation (wavelength 1.54 Å) at 30 kV and 10 mA in θ–2θ geometry. The increment was fixed at 0.05°. The chitosan samples were placed on a silicon single crystal specially cut to provide a low background free from any interfering diffraction peaks. The XRD results on the replicate samples of each fabricated material were almost identical, with <10% variation. The intensity values were only subtracted from the blank run intensity of the sample holder and presented.

##### Atomic Force Microscopy

Surface morphology and roughness of the chitosan were accessed by atomic force microscopy (AFM; Park Systems NX20, Republic of Korea). AFM was operated in a non-contact mode in air. A PPP-NCHR probe (Park Systems, Suwon, Republic of Korea) with a nominal resonance frequency of 330 kHz and force constant of 42 N m^−1^ was used. AFM images of 5 × 5 μm^2^ representative areas on the chitosan samples were acquired with a scan rate of 0.6–0.8 Hz to gain >90% matching of backward and forward scans. The surface roughness parameter (Rq) was estimated using the Park Systems XEI 1.8.5 image analysis software.

##### TENG Testing

The TENG setup is displayed in Figure 12a. The Chitosan films were attached to a copper tape (acting as electrode) and then fastened on a linear motor as one tribolayer, and the counter-tribolayers were fixed on a copper tape and mounted on the linear motor according to the method reported by Zhang et al. (2020) [3]. PTFE is often used as a reference material in TENG studies; therefore, it was used as the counter-tribolayer in this study. The thickness of the PTFE film was 130 µm. The chitosan film thickness was determined from the average of ten measurements using a micrometre screw gauge. The contact-separation speed of the TENGs was set to 0.3 m s^−1^ and the size of the TENGs was 3 × 3 cm^2^. Electrical signals were measured with a PXI4071 digital multimeter (National Instruments Corp, Austin, TX, USA).

The prepared chitosan films were conditioned at a temperature of 40 °C for 24 h to equilibrate the moisture content in the samples before the TENG measurements and the characterisations.

## Figures and Tables

**Table 1:** Ostwald–de Waele fitting parameters for chitosan solutions in aqueous solutions of AA.

Sample	K (Pa s^n^)	*n*	r
1.0 wt.% low-MW chitosan in 5.0 wt.% AA aq.	0.04	0.96	0.997
1.0 wt.% high-MW chitosan in 5.0 wt.% AA aq.	0.31	0.85	0.988
2.5 wt.% low-MW chitosan in 0.5 wt.% AA aq.	0.07	0.95	0.990
2.5 wt.% low-MW chitosan in 1.0 wt.% AA aq.	0.08	0.95	0.996
2.5 wt.% low-MW chitosan in 2.0 wt.% AA aq.	0.45	0.93	0.986
2.5 wt.% low-MW chitosan in 5.0 wt.% AA aq.	0.55	0.86	0.991
2.5 wt.% low-MW chitosan in 10.0 wt.% AA aq.	0.50	0.87	0.991
2.5 wt.% high-MW chitosan in 0.5 wt.% AA aq.	2205	1.03	0.985
2.5 wt.% high-MW chitosan in 1.0 wt.% AA aq.	38.5	0.64	0.977
2.5 wt.% high-MW chitosan in 2.0 wt.% AA aq.	14.2	0.56	0.999
2.5 wt.% high-MW chitosan in 5.0 wt.% AA aq.	40.9	0.33	0.997
2.5 wt.% high-MW chitosan in 10.0 wt.% AA aq.	29.2	0.49	0.987

**Table 2:** The triboelectric data, including standard deviation from 10 peaks of each measurement.

Sample	Current (µA)	Voltage (V)
low-MW 2 wt.%	107.5 ± 0.3	188.8 ± 0.7
low-MW 5 wt.%	108.1 ± 0.3	223.6 ± 0.4
low-MW 10 wt.%	110.8 ± 0.9	255.7 ± 1.1
high-MW 2 wt.%	142.8 ± 0.3	259.8 ± 1.1
high-MW 5 wt.%	150.1 ± 0.3	271.4 ± 1.3
high-MW 10 wt.%	178.4 ± 0.4	291.9 ± 1.0

## Data Availability

The data is contained within the article.
